# Pharmacogenomics of statin-related myopathy: Meta-analysis of rare variants from whole-exome sequencing

**DOI:** 10.1371/journal.pone.0218115

**Published:** 2019-06-26

**Authors:** James S. Floyd, Katarzyna M. Bloch, Jennifer A. Brody, Cyrielle Maroteau, Moneeza K. Siddiqui, Richard Gregory, Daniel F. Carr, Mariam Molokhia, Xiaoming Liu, Joshua C. Bis, Ammar Ahmed, Xuan Liu, Pär Hallberg, Qun-Ying Yue, Patrik K. E. Magnusson, Diane Brisson, Kerri L. Wiggins, Alanna C. Morrison, Etienne Khoury, Paul McKeigue, Bruno H. Stricker, Maryse Lapeyre-Mestre, Susan R. Heckbert, Arlene M. Gallagher, Hector Chinoy, Richard A. Gibbs, Emmanuelle Bondon-Guitton, Russell Tracy, Eric Boerwinkle, Daniel Gaudet, Anita Conforti, Tjeerd van Staa, Colleen M. Sitlani, Kenneth M. Rice, Anke-Hilse Maitland-van der Zee, Mia Wadelius, Andrew P. Morris, Munir Pirmohamed, Colin A. N. Palmer, Bruce M. Psaty, Ana Alfirevic, on behalf of the PREDICTION-ADR Consortium and EUDRAGENE

**Affiliations:** 1 Department of Medicine, University of Washington, Seattle, Washington, United States of America; 2 MRC Centre for Drug Safety Science, Department of Molecular and Clinical Pharmacology, University of Liverpool, Liverpool, United Kingdom; 3 Division of Molecular and Clinical Medicine, University of Dundee, Dundee, United Kingdom; 4 Functional and Comparative Genomics, Institute of Integrative Biology, University of Liverpool, Liverpool, United Kingdom; 5 School of Population Health and Environmental Sciences, London, United Kingdom; 6 Human Genetics Center, University of Texas Health Science Center, Houston, United States of America; 7 Medical School, University of Liverpool, Liverpool, United Kingdom; 8 Department of Medical Sciences, Clinical Pharmacology and Science for Life Laboratory, Uppsala University, Uppsala University Hospital, Uppsala, Sweden; 9 Medical Products Agency, Uppsala, Sweden; 10 Swedish Twin Registry, Department of Medical Epidemiology and Biostatistics, Karolinska Institutet, Stockholm, Sweden; 11 Clinical Lipidology and Rare Lipid Disorders Unit, Department of Medicine, Université de Montréal Community Gene Medicine Center, Lipid Clinic Chicoutimi Hospital and ECOGENE-21 Clinical and Translational Research Center, Chicoutimi, Quebec, Canada; 12 Human Genetics Center, Department of Epidemiology, Human Genetics, and Environmental Sciences, School of Public Health, The University of Texas Health Science Center at Houston, Houston, Texas, United States of America; 13 Usher Institute of Population Health Sciences and Informatics, University of Edinburgh Medical School, Edinburgh, Scotland, United Kingdom; 14 Department of Epidemiology, Erasmus Medical Centre, Rotterdam, the Netherlands; 15 Paul Sabatier University - Toulouse III, UPS Toulouse, Laboratoire de Pharmacologie Medicale et Clinique, Toulouse, France; 16 Department of Epidemiology, University of Washington, Seattle, Washington, United States of America; 17 Clinical Practice Research Datalink (CPRD) Medicines and Healthcare Products Regulatory Agency, London, United Kingdom; 18 Rheumatology Department, Salford Royal NHS Foundation Trust, Manchester Academic Health Science Centre, Salford, United Kingdom; 19 Human Genome Sequencing Center, Baylor College of Medicine, Houston, United States of America; 20 Centre Hospitalier Universitaire de Toulouse, CHU Toulouse, Centre de Pharmacovigilance, Toulouse, France; 21 Departments of Pathology & Laboratory Medicine and Biochemistry, Larner College of Medicine, University of Vermont, Burlington, Vermont, United States of America; 22 U.O. Farmacologia, Policlinico "Gb Rossi", Verona, Italy; 23 Division of Informatics, Imaging & Data Sciences, University of Manchester, Manchester, United Kingdom; 24 Department of Biostatistics, University of Washington, Seattle, Washington, United States of America; 25 Respiratory Medicine/Pediatric Respiratory Medicine, AMC, Amsterdam, the Netherlands; 26 Department of Biostatistics, University of Liverpool, Liverpool, United Kingdom; University of Tampere, FINLAND

## Abstract

**Aims:**

Statin-related myopathy (SRM), which includes rhabdomyolysis, is an uncommon but important adverse drug reaction because the number of people prescribed statins world-wide is large. Previous association studies of common genetic variants have had limited success in identifying a genetic basis for this adverse drug reaction. We conducted a multi-site whole-exome sequencing study to investigate whether rare coding variants confer an increased risk of SRM.

**Methods and results:**

SRM 3–5 cases (N = 505) and statin treatment-tolerant controls (N = 2047) were recruited from multiple sites in North America and Europe. SRM 3–5 was defined as symptoms consistent with muscle injury and an elevated creatine phosphokinase level >4 times upper limit of normal without another likely cause of muscle injury. Whole-exome sequencing and variant calling was coordinated from two analysis centres, and results of single-variant and gene-based burden tests were meta-analysed. No genome-wide significant associations were identified. Given the large number of cases, we had 80% power to identify a variant with minor allele frequency of 0.01 that increases the risk of SRM 6-fold at genome-wide significance.

**Conclusions:**

In this large whole-exome sequencing study of severe statin-related muscle injury conducted to date, we did not find evidence that rare coding variants are responsible for this adverse drug reaction. Larger sample sizes would be required to identify rare variants with small effects, but it is unclear whether such findings would be clinically actionable.

## Introduction

Lipid-lowering drugs that inhibit 5-hydroxy-3-methylglutaryl-coenzyme A (HMG-CoA), known as statins, are widely used for the primary and secondary prevention of cardiovascular disease (CVD). All statins can cause muscle toxicity that ranges in severity from mild muscle pain to severe myopathy or rhabdomyolysis, which can lead to kidney failure and death [1]. The most severe forms of statin-related muscle injury are uncommon, suggesting a genetic predisposition. For instance, severe statin-related myopathy (SRM) restricted to cases with creatine phosphokinase (CK) levels >10x the upper limit of normal (ULN) (SRM 4) requiring hospitalisation, occurs in approximately 1–10 out of every 10,000 individuals taking standard statin doses [2].

Previous genome-wide association studies (GWAS) have identified a common variant in the drug transporter gene *SLCO1B1* that increases the risk of SRM defined by varying CK elevation criteria, 2- to 4-fold through a pharmacokinetic mechanism, but the underlying causes of this adverse drug reaction remain largely unexplained [3–5]. To investigate whether rare variants in coding regions of the genome may cause SRM, we conducted whole-exome sequencing on cases of SRM identified from multiple sites throughout North America and Europe, harmonized variant calling and analysis at two centres, and meta-analysed results from single-variant and gene-based burden tests across two large case-control studies.

## Materials and methods

### Study population and overall study design

Identification and recruitment of case and control subjects, whole-exome sequencing, variant calling, and statistical analyses were coordinated by two analysis centers: one based at the University of Washington that included 291 cases and 1540 controls from North America and the United Kingdom (“US-UK”) and one based in Liverpool that included 214 cases and 507 controls from Europe (“PREDICTION-ADR”). Within each of these two large case-control studies, participants were recruited from multiple sites. Because there were few non-European ancestry participants in either case-control study, and many sites had none, we restricted analyses to participants of European ancestry. The details of recruitment at each site are described in the **Supporting Information files**. Whole-exome sequencing and variant calling were harmonized within each of the two case-control studies, and investigators from both studies agreed prospectively to implement a coordinated statistical analysis plan, which included protocols for meta-analysis of results from both single-variant and gene-based burden tests. The University of Washington Human Subjects Division, East of Scotland Research Ethics Service REC1 and REC2, North West–Liverpool Central Research Ethics Committee, Regional Ethical Review Boards in Uppsala (2008/213 and 2010/231) and Stockholm (2007/644-31 and 2011/463-32) and the Medical Ethics Committee from the University Medical Center in Utrecht reviewed and approved this study.

### Case definition

Using a previously published case definition (S1 Table), SRM 3–5 cases received statin treatment at the time of onset of muscle injury symptoms, had a peak CK level > 4x ULN (SRM 3) or supporting clinical evidence of rhabdomyolysis (SRM 5), and had no other likely cause of muscle injury other than statin treatment.[1] In each study, non-statin causes of muscle injury were excluded based on a review of medical records and/or interview of patients by physicians or study staff. Severe SRM cases (SRM 4) sometimes called severe myopathy and SRM 5, sometimes called rhabdomyolysis in some previous studies [6], met these criteria and had a peak CK level > 10x ULN (S1 Table). SRM cases with myopathy and evidence of anti-3-hydroxy-3-methyl-glutaryl-coenzyme A reductase (HMGCR) autoantibodies (SRM 6), which are suggestive of autoimmune myopathy, were excluded at all sites [7]. In both case-control studies, statin treatment-tolerant controls subjects were users of statins who had no evidence of muscle injury during follow up.

### Whole-exome sequencing, variant calling, quality control

Exome sequencing, variant calling, and quality control of sequence data were performed separately in each case-control study (S1 Appendix and S2 Table). Each study conducted sequencing and called variants for cases and controls together, to eliminate batch effects across case-control sampling. We created a combined variant annotation file including all quality-controlled variant sites observed in either study. Variants were annotated using ANNOVAR [8] and dbNSFP v3.0 [9] according to the reference genome GRCh37.

### Statistical analysis

Within each case-control study, which included only European ancestry participants, population structure was adjusted for using principal components derived from a genetic relationship matrix. To estimate associations between single variants and the risk of SRM, the Firth bias-corrected score test was used [10]. The primary single-variant analysis included all SRM cases and all control subjects. Single variant analyses were restricted to variants with at least 10 copies of the minor allele, but not further restricted by annotated variant function. Results from the two case-control studies were combined using a Z-based meta-analysis [11]. The following secondary analyses were also conducted: restriction to users of simvastatin, atorvastatin, and cerivastatin (the three most commonly-used statins in these studies); stratification of SRM cases into moderate (SRM 3: CK ≥ 4x ULN and <10x ULN) and severe (SRM 4 and 5: CK ≥ 10x ULN) categories; and inclusion of all missense variants. In the US-UK case-control study, information on fibrate use was collected. Because fibrates can cause drug-drug interactions with statins through inhibition of the metabolism of statins, and because strong drug-drug interactions can mask the presence of a genuine drug-gene interaction that may be less potent, we conducted secondary analyses restricted to non-users of fibrates.

Rare variants (MAF < 1%) were collapsed into gene-burden scores and tested for association with the risk of SRM [12]. Gene-burden results from the two case-control studies were combined using a Z-based meta-analysis. The primary gene-based analysis included variants with MAF < 1% that are missense, bioinformatically predicted to be damaging (MetaLR > .5) [13], stop-gain/loss, coding indels, or splice site variants. Secondary gene-burden analyses restricted to users of simvastatin or atorvastatin, stratified SRM cases into moderate (SRM 3: CK > = 4x ULN and <10x ULN) and severe (SRM 4 and 5: CK > = 10x ULN) categories, and included rare missense variants regardless of their prediction score for function.

For each analysis, the Bonferroni-corrected threshold for statistical significance was P = 0.05 / number of single variants or genes tested. For the primary single-variant analysis, we conducted *post-hoc* power calculations using QUANTO 1.1 [14, 15] to determine study power in the primary analysis population across a range of effect sizes and MAFs, based on α = 0.05 / number of single variants included in the primary analysis and an estimated disease probability among control subjects of 0.0001 [16].

The full results from each meta-analysis will be available on dbGaP within the next release of Cohort for Heart and Aging Research in Genomic Epidemiology Results CHARGE Consortium summary results (accession phs000930) (http://www.chargeconsortium.com/main/results). Information on how to access individual-level study data is provided in the Supporting Information files.

## Results

Across the two case-control studies, there were 505 SRM cases and 2,047 treatment-tolerant controls that passed sample-level quality control (Table 1). Most of the US-UK cases (mean CK 159x ULN) were severe (65%) and used cerivastatin or simvastatin, while most of the PREDICTION-ADR cases (mean CK 32x ULN) were moderate (61%) and used simvastatin or atorvastatin.

**Table 1:** Characteristics of statin-related myopathy cases and statin-tolerant controls from two international case-control studies.

	US-UK Cases N = 291	US-UK Controls N = 1540	PREDICTION-ADR Cases N = 214	PREDICTION-ADR Controls N = 507
**Demographic data**				
Mean age, years (SD)	65 (10.4)	62 (8.3)	66 (11)	69 (9.5)
Age range, years	[34, 91]	[44, 87]	[21, 92]	[39, 93]
Gender, % female	44.0%	53.8%	34.1%	36.5%
**Statin Type, %**				
Atorvastatin	32 (11%)	121 (8%)	57 (27%)	129 (25%)
Cerivastatin	146 (50%)	10 (1%)	1 (0%)	0
Fluvastatin	3 (1%)	1 (0%)	4 (2%)	4 (1%)
Pravastatin	7 (2%)	172	13 (6%)	26 (5%)
Rosuvastatin	8 (3%)	130 (12%)	12 (6%)	7 (1%)
Simvastatin	93 (33%)	82 (53%)	127 (59%)	341 (67%)
Lovastatin	0	28 (2%)	0	0
Unknown type	2 (1%)	996 (65%)	0	0
CK/ULN, mean (SD)	159 (325)	N/A	32 (74)	N/A
CK/ULN, range	[4, 529]	N/A	[4, 542]	N/A
**Myopathy type**				
Moderate (SRM 3)	101 (35%)	N/A	132 (61%)	N/A
Severe (SRM 4 or 5)	190 (65%)	N/A	82 (39%)	N/A
Severe, no fibrates	98 (34%)	N/A	53 (25%)	N/A

### Single-variant results

**Fig 1:**
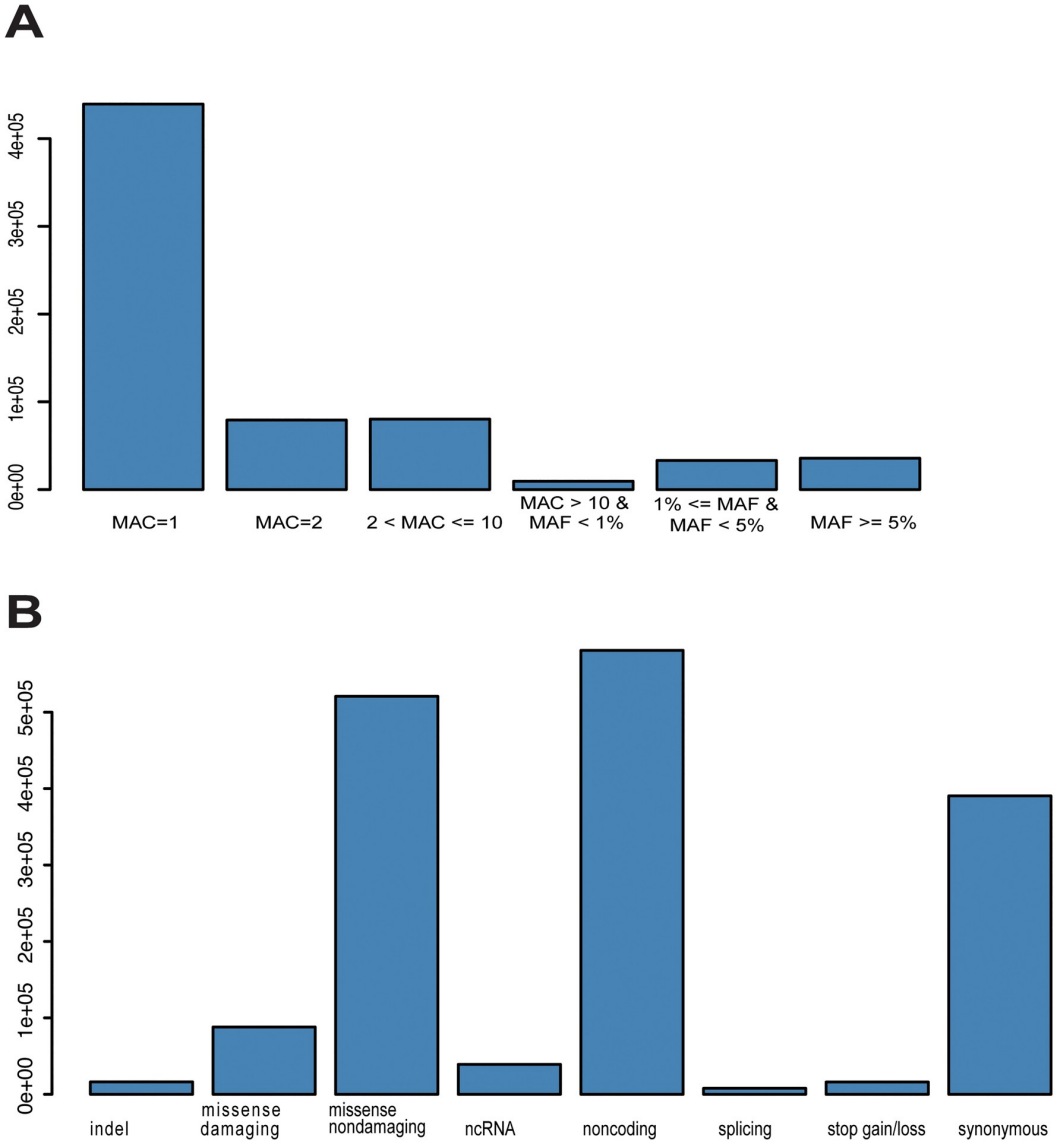
Single variant analysis. Single variants included in primarily analysis by frequency (panel A) and type (panel B).

The mean sequencing depth in US-UK case-control study was 78x, and in PREDICTION-ADR it was 56x. After restricting to rare (MAF < 0.01) likely damaging variants, 162,813 variants passed quality control in one or both studies and were included in meta-analyses (Fig 1). In primary analyses that included all SRM cases and all statin-tolerant controls, there were no variants that met the Bonferroni-corrected threshold for statistical significance (P < 3.07 x 10^−7^) (Table 2). The widely replicated locus for statin-related muscle injury in *SLCO1B1* (rs4149056) was associated with a 1.59-fold increased risk of SRM (95% confidence interval, 1.41–1.77), but this finding was not significant after correction for the number of single variants tested.

**Table 2:** Top associations from meta-analysis of primary single-variant results from US-UK and PREDICTION-ADR case-control studies of statin-related myopathy.

Ch:position	Coded/referent allele	MAF	P value	US-UK Beta (SE)	PREDICTION Beta (SE)	Annotation
6:44221262	G/A	0.6%	4.59E-07	2.89 (0.60)	NA	Nonsynonymous:HSP90AB1
22:20117046	C/T	4.3%	1.04E-06	1.00 (0.24)	0.66 (0.24)	Intergenic
12:21331549	C/T	17.8%	1.26E-06	0.59 (0.12)	0.25 (0.15)	Nonsynonymous:SLCO1B1
17:4337283	A/G	16.8%	1.84E-06	-0.68 (0.15)	NA	Synonymous:SPNS3
6:30918065	C/T	0.6%	5.35E-06	2.23 (0.48)	NA	Synonymous:DPCR1
6:42236725	GCT/G	0.3%	6.95E-06	3.08 (0.74)	NA	Frameshift:TRERF1
7:21778429	C/T	5.5%	1.79E-05	-0.845 (0.26)	-0.73 (0.30)	Nonsynonymous:DNAH11
7:36552656	A/G	37.9%	2.49E-05	NA	-0.55 (0.13)	Utr3:AOAH
6:32261014	A/C	1.4%	2.67E-05	1.02 (0.37)	1.44 (0.45)	Nonsynonymous:C6orf10

MAF = minor (coded) allele frequency

Secondary analyses restricted by statin type and stratified by moderate or severe cases of SRM did not yield any associations that met criteria for statistical significance (S3 Table). Among non-users of fibrates in the US-UK case-control study, the *SLCO1B1* variant rs4149056 was associated with a 4.01-fold increased risk of SRM (95% confidence interval, 2.61–6.17), and this association met criteria for statistical significance (P = 5.46 x 10^−11^) (S4 Table).

### Gene-burden results

In total, 10,244 genes passed quality control thresholds in one or both studies and were included in the meta-analyses of burden test results. In primary analyses of all SRM cases and all controls, no genes met the Bonferroni-corrected threshold for statistical significance (P < 4.9 x 10^−6^) (Table 3). Similarly, none of the secondary burden tests analyses yielded genome-wide significant findings (S5 Table).

**Table 3:** Top associations from meta-analysis of primary gene burden results from US-UK and PREDICTION-ADR case-control studies of statin-related myopathy.

Gene	cMAF	P value	US-UK Beta (SE)	PREDICTION Beta (SE)
TRERF1	0.5%	0.00017	2.78 (0.63)	0.98 (2.31)
FOXP4	0.8%	0.00066	1.31 (0.39)	1.22 (1.03)
BAAT	1.3%	0.0011	0.72 (0.34)	1.19 (0.47)
EPB41	1.8%	0.0014	0.69 (0.25)	1.15 (0.67)
ESYT3	3.5%	0.0024	0.62 (0.20)	0.46 (0.35)
NR4A3	2.2%	0.0028	0.41 (0.34)	1.12 (0.40)
TMEM239	0.6%	0.0032	1.89 (0.79)	0.98 (0.56)
FAN1	2.8%	0.0035	0.52 (0.22)	1.09 (0.63)
MYBPH	0.9%	0.0035	1.15 (0.38)	0.68 (0.84)

cMAF = cumulative minor allele frequency

### Post-hoc power calculations

**Fig 2:**
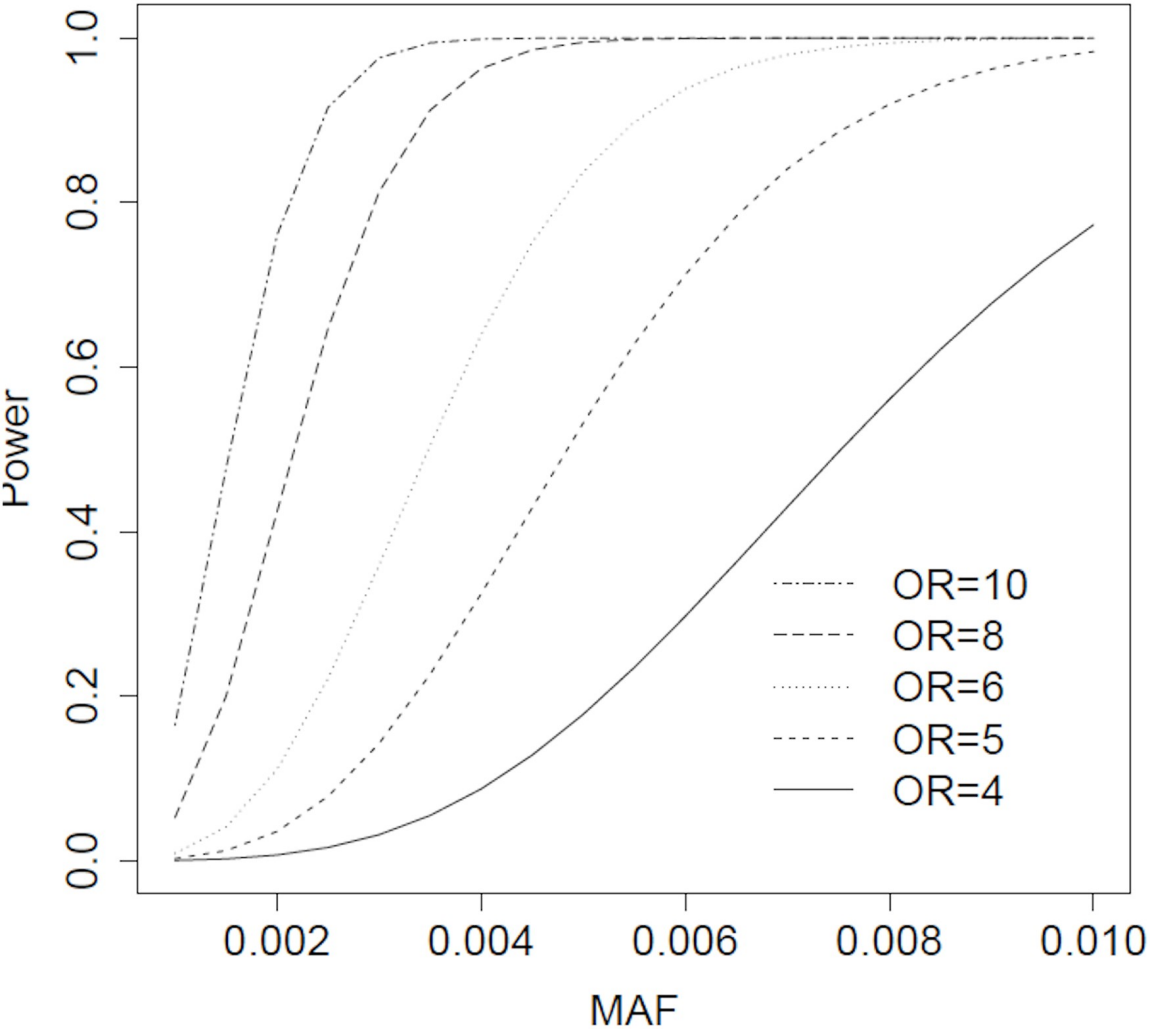
Power calculation. *Post-hoc* power calculations to identify a single variant associated with statin-related myopathy in primary analysis population given effect size (odds ratio) and minor allele frequency (MAF), α = 0.05 / 162,813.

We conducted *post-hoc* power calculations to estimate study power in the primary analysis population to identify single-variants associated with SRM given a range of MAFs and effect sizes (Fig 2). For a variant with a MAF of 0.01, we had 98% power to detect an OR of 5, and for a variant with a MAF of 0.005, we had 84% power to detect an OR of 6.

## Discussion

We recruited more than 500 patients with statin-related muscle injury from multiple sites in North America and Europe to conduct the largest whole-exome sequencing study of this adverse drug reaction to date. Using high-throughput sequencing technology, joint variant calling at two experienced analysis centres, and rigorous statistical methods, we did not identify any novel coding variants associated with SRM 3–5. We also evaluated hypotheses about the burden of rare coding variants within genes, muscle injury due to specific statin drugs, and the severity of muscle injury; the findings from these analyses were null as well.

In 2008, Link *et al*. found that a common nonsynonymous variant in the drug transporter *SLCO1B1* (rs4149056) was associated with a 4-fold increase in the risk of myopathy among users of high dose simvastatin at genome-wide levels of statistical significance [4]. This finding has been widely replicated in other settings for other statin drugs [3, 5, 17–19], and in our study this common coding variant was associated with a 4-fold increased risk of SRM 5 among non-users of fibrates. Interestingly, we did not find an association between the *SLCO1B1* polymorphisms and mild forms of SRM. Attempts to discover additional common variants related to statin-related muscle injury have failed to yield replicable findings [20–22]. A recent study from the Czech Republic conducted whole-exome sequencing on 88 patients with mild statin-associated muscle toxicity, reporting an increased burden of rare variation in 24 genes [23]. We did not replicate these findings in our study, perhaps due to different phenotypes as most of our patients have more severe SRM. The validity of these findings may be limited because of the use of publicly available sequencing repositories that do not have information on medication use as the referent group rather than statin-tolerant control subjects.

Strengths of our study include the recruitment of patients from multiple international sites; the use of a standardized case-definition for different forms of myopathy in our study (SRM 3–5) with the mildest being CK>4x ULN (SRM 3) and rhabdomyolysis ranging from CK>10 to >40xULN with end-organ impairment and muscle symptoms[1], the depth of whole-exome sequencing in both case-control studies; the use of statin exposed controls to minimize genetic associations with indications for statin therapy, the coordinated statistical analysis plan with correction for multiple testing to reduce the risk of false positive findings; and the prospective plan for a meta-analysis of results across two large-case control studies.

There were several limitations. As with any adverse drug reaction, it is difficult to attribute causality to a specific drug, even with prospectively developed case definitions and expert adjudication of cases. In addition, the presence of unknown drug-drug interactions with statin that increase the risk of SRM may have masked our ability to identify genuine drug-gene interactions. We recognize that our inability to recruit a large number of cases from diverse ancestries limits the generalizability of our findings to variants observed in European ancestry participants. Half of the SRM cases in the US-UK case-control study were due to the drug cerivastatin, and we were unable to identify a large number of controls subjects with WES who used this drug, since it was removed from the market in 2001. If there is heterogeneity in the effects of genetic variants on the risk of SRM by the type of statin drug or severity of SRM, this might have reduced our overall power to identify genetic loci for this adverse drug reaction in our primary analysis, which included all samples. Finally, using our stringent correction for multiple comparisons, we may have missed an association with rare, loss of function variants in biologically plausible metabolising enzyme or transporter genes that could have led to large increase in drug exposure and therefore muscle damage in some individuals [24].

While our post-hoc calculations demonstrated that our study was well-powered to identify rare coding variants (MAF 1%) with large effect sizes (OR 5), we had limited power to detect rare variants with small- or moderate-sized effects, or to detect even rarer variants with large effects. For instance, to have 90% power to identify a coding variant with a frequency of 0.001 among controls that increases the risk of SRM 6-fold, we would have to recruit and conduct whole-exome sequencing on approximately 2,700 cases with this rare adverse drug reaction, matched with 10,800 treatment-tolerant controls.

It is possible that rare variants exist that increase the risk of SRM, perhaps in non-coding regions of the genome, but they are unlikely to be detected with the sample sizes available in ongoing studies. Based on the null findings from our study, genotyping rare coding alleles for the prediction of severe statin-related muscle injury seems unlikely to yield clinically actionable findings.

## Supporting information

S1 Appendix Recruitment and study populations; genotyping and variant calling. (DOCX)

S1 Table Definition of statin-related myopathy. (DOCX)

S2 Table Whole exome sequence metrics, PREDICTION-ADR. (DOCX)

S3 Table Top hits from single variant meta-analysis. Primary analysis including all samples and analyses stratified by statin type and severity of statin-related myopathy.
(XLSX)

S4 Table Top hits from single variant meta-analysis, non-users of fibrates in the US-UK case-control study. *SLCO1B1* variant rs4149056 (12:21331549:T:C) was associated with a 4.01-fold increased risk of SRM (95% CI, 2.61–6.17), and this association met criteria for statistical significance (P = 5.46 x 10^−11^).
(XLSX)

S5 Table Top hits from gene burden meta-analysis. Primary analysis including all samples and secondary analyses stratified by statin type and severity of statin-related myopathy.
(XLSX)

S1 Fig QQ plots of meta-analysed single-variant results from US-UK and PREDICTION-ADR case-control studies for primary and secondary analyses. (DOCX)

S2 Fig QQ plots of meta-analysed gene burden test results from US-UK and PREDICTION-ADR case-control studies for primary and secondary analyses. Columns: (1) predicted damaging variants, (2) all loss of function, nonsynonymous and missense. Rows: (1) all SRM, (2) simvastatin only, (3) atorvastatin only, (4) mild SRM, (5) severe SRM.
(PDF)

## References

[1] Alfirevic A, Neely D, Armitage J, Chinoy H, Cooper RG, Laaksonen R, et al Phenotype standardization for statin-induced myotoxicity. Clin Pharmacol Ther. 2014;96(4):470–6. 10.1038/clpt.2014.121 . 24897241 PMC4172546

[2] Graham DJ, Staffa JA, Shatin D, Andrade SE, Schech SD, La Grenade L, et al Incidence of hospitalized rhabdomyolysis in patients treated with lipid-lowering drugs. JAMA. 2004;292(21):2585–90. 10.1001/jama.292.21.2585 . 15572716

[3] Carr DF, O’Meara H, Jorgensen AL, Campbell J, Hobbs M, McCann G, et al SLCO1B1 Genetic Variant Associated With Statin-Induced Myopathy: A Proof-of-Concept Study Using the Clinical Practice Research Datalink. Clin Pharmacol Ther. 2013;94(6):695–701. 10.1038/clpt.2013.161 . 23942138 PMC3831180

[4] Link E, Parish S, Armitage J, Bowman L, Heath S, Matsuda F, et al SLCO1B1 variants and statin-induced myopathy—a genomewide study. The New England journal of medicine. 2008;359(8):789–99. Epub 2008/07/25. 10.1056/NEJMoa0801936 . 18650507

[5] Marciante KD, Durda JP, Heckbert SR, Lumley T, Rice K, McKnight B, et al Cerivastatin, genetic variants, and the risk of rhabdomyolysis. Pharmacogenetics and genomics. 2011;21(5):280–8. 10.1097/FPC.0b013e328343dd7d . 21386754 PMC3076530

[6] Floyd JS, Kaspera R, Marciante KD, Weiss NS, Heckbert SR, Lumley T, et al A screening study of drug-drug interactions in cerivastatin users: an adverse effect of clopidogrel. Clin Pharmacol Ther. 2012;91(5):896–904. 10.1038/clpt.2011.295 . 22419147 PMC3830936

[7] Mammen AL, Chung T, Christopher-Stine L, Rosen P, Rosen A, Doering KR, et al Autoantibodies against 3-hydroxy-3-methylglutaryl-coenzyme A reductase in patients with statin-associated autoimmune myopathy. Arthritis Rheum. 2011;63(3):713–21. 10.1002/art.30156 . 21360500 PMC3335400

[8] Wang K, Li M, Hakonarson H. ANNOVAR: functional annotation of genetic variants from high-throughput sequencing data. Nucleic Acids Res. 2010;38(16):e164 10.1093/nar/gkq603 . 20601685 PMC2938201

[9] Liu X, Wu C, Li C, Boerwinkle E. dbNSFP v3.0: A One-Stop Database of Functional Predictions and Annotations for Human Nonsynonymous and Splice-Site SNVs. Human mutation. 2016;37(3):235–41. 10.1002/humu.22932 . 26555599 PMC4752381

[10] Ma C, Blackwell T, Boehnke M, Scott LJ, Go TDi. Recommended joint and meta-analysis strategies for case-control association testing of single low-count variants. Genetic epidemiology. 2013;37(6):539–50. 10.1002/gepi.21742 . 23788246 PMC4049324

[11] Willer CJ, Li Y, Abecasis GR. METAL: fast and efficient meta-analysis of genomewide association scans. Bioinformatics. 2010;26(17):2190–1. Epub 2010/07/10. 10.1093/bioinformatics/btq340 . 20616382 PMC2922887

[12] Li B, Leal SM. Methods for detecting associations with rare variants for common diseases: application to analysis of sequence data. Am J Hum Genet. 2008;83(3):311–21. 10.1016/j.ajhg.2008.06.024 . 18691683 PMC2842185

[13] Dong C, Wei P, Jian X, Gibbs R, Boerwinkle E, Wang K, et al Comparison and integration of deleteriousness prediction methods for nonsynonymous SNVs in whole exome sequencing studies. Hum Mol Genet. 2015;24(8):2125–37. 10.1093/hmg/ddu733 . 25552646 PMC4375422

[14] Gauderman WJ. Sample size requirements for matched case-control studies of gene-environment interaction. Stat Med. 2002;21(1):35–50. Epub 2002/01/10. . 11782049 10.1002/sim.973

[15] Gauderman WJ, Morrison JM. QUANTO 1.1: A computer program for power and sample size calculations for genetic-epidemiology studies, http://hydra.usc.edu/gxe, 2006.

[16] Graham DJ, Staffa JA, Shatin D, Andrade SE, Schech SD, La Grenade L, et al Incidence of hospitalized rhabdomyolysis in patients treated with lipid-lowering drugs. JAMA: the journal of the American Medical Association. 2004;292(21):2585–90. Epub 2004/12/02. 10.1001/jama.292.21.2585 . 15572716

[17] Brunham LR, Lansberg PJ, Zhang L, Miao F, Carter C, Hovingh GK, et al Differential effect of the rs4149056 variant in SLCO1B1 on myopathy associated with simvastatin and atorvastatin. The pharmacogenomics journal. 2012;12(3):233–7. 10.1038/tpj.2010.92 . 21243006

[18] Linde R, Peng L, Desai M, Feldman D. The role of vitamin D and SLCO1B1*5 gene polymorphism in statin-associated myalgias. Dermato-endocrinology. 2010;2(2):77–84. 10.4161/derm.2.2.13509 . 21547103 PMC3081682

[19] Voora D, Shah SH, Spasojevic I, Ali S, Reed CR, Salisbury BA, et al The SLCO1B1*5 genetic variant is associated with statin-induced side effects. Journal of the American College of Cardiology. 2009;54(17):1609–16. 10.1016/j.jacc.2009.04.053 . 19833260 PMC3417133

[20] Carr DF, Alfirevic A, Johnson R, Chinoy H, van Staa T, Pirmohamed M. GATM gene variants and statin myopathy risk. Nature. 2014;513(7518):E1 Epub 2014/09/19. 10.1038/nature13628 . 25230669

[21] Floyd JS, Bis JC, Brody JA, Heckbert SR, Rice K, Psaty BM. GATM locus does not replicate in rhabdomyolysis study. Nature. 2014;513(7518):E1–3. Epub 2014/09/19. 10.1038/nature13629 . 25230668 PMC4230441

[22] Mangravite LM, Engelhardt BE, Medina MW, Smith JD, Brown CD, Chasman DI, et al A statin-dependent QTL for GATM expression is associated with statin-induced myopathy. Nature. 2013;502(7471):377–80. Epub 2013/09/03. 10.1038/nature12508 . 23995691 PMC3933266

[23] Neroldova M, Stranecky V, Hodanova K, Hartmannova H, Piherova L, Pristoupilova A, et al Rare variants in known and novel candidate genes predisposing to statin-associated myopathy. Pharmacogenomics. 2016;17(13):1405–14. 10.2217/pgs-2016-0071 . 27296017

[24] Teslovich TM, Musunuru K, Smith AV, Edmondson AC, Stylianou IM, Koseki M, et al Biological, clinical and population relevance of 95 loci for blood lipids. Nature. 2010;466(7307):707–13. 10.1038/nature09270 . 20686565 PMC3039276

