# Supplementary material for: Pharmacogenomics of statin-related myopathy: Meta-analysis of rare variants from whole-exome sequencing
Source: PLoS One. 2019 Jun 26;14(6):e0218115. doi: 10.1371/journal.pone.0218115 (PMC6594672; doi:10.1371/journal.pone.0218115)
Supplement: S1 Appendix — (DOCX) [file pone.0218115.s001.docx]

**Recruitment and study populations: North America and United Kingdom (“US-UK”)**

Rhabdomyolysis cases from the University of Washington (n=146) were recruited through attorneys who had represented cerivastatin users with rhabdomyolysis.^1, 2^ Case subjects who participated in the study were demographically similar to those who did not. For consenting cases, study staff conducted a telephone interview and obtained copies of medical records from attorneys, physicians, and hospitals. Trained abstracters used medical records to validate rhabdomyolysis events, collect information about participants’ medical history, and exclude other potential etiologies of muscle injury. Rhabdomyolysis was defined as muscle pain or weakness, and a creatine kinase (CK) level > 10 times the upper limit of normal (ULN). For participants who provided written consent for sharing of de-identified individual-level genotype and phenotype data, these data will be made available on dbGaP.

Statin-induced myopathy cases from the University of Liverpool (n=124) were recruited through the United Kingdom Clinical Practice Research Datalink (CPRD, http://www.cprd.com) from a cohort of ~600,000 patients receiving statins.^3, 4^ Participation was restricted to Caucasians ≥18 years of age and with the first-ever statin prescription at least 1 year after the start of CPRD data collection. Potential cases were selected from the database if they discontinued their implicated statin therapy and demonstrated an increase in CK > 4x ULN. General practitioners were contacted with a list of potential cases from their practices. After being given the opportunity to decline involvement, they were first asked to review the list and remove any patients they considered unsuitable. They were then asked to contact suitable patients by letter requesting participation. Consenting case patients provided either a saliva sample (by post) or a blood sample (by visit to the practice). All samples were then forwarded to the University of Liverpool for processing. To preserve anonymity, patient and practice identifier codes were used throughout the recruitment process, and all patient contact was through the general practitioner only. An additional 5 cases of statin-induced myopathy that conformed to the above Liverpool criteria were included from the tertiary adult muscle clinic run through Salford Royal NHS Foundation Trust, UK, and recruited via the UKMYONET genetic study. Participants from this study did not provide consent for investigators to share their individual-level study data.

Statin-induced myopathy and rhabdomyolysis adult cases (≥ 18 years old) from the EUDRAGENE^5^ collaboration were recruited through identification from pharmacovigilance reports and national EHRs of drug utilization and morbidity in primary care and hospitals. Detailed case definitions were developed, based on consultation with specialists with appropriate exclusion criteria and reviewed for causality using WHO criteria. Rhabdomyolysis was defined as muscle pain or weakness, and a creatine kinase (CK) level > 10 times the sex-standardised upper limit of normal (ULN). Further medical history relevant to the diagnosis and to exclude other causes of myopathy was sought from the patient’s responsible physician with their consent. Consenting case patients provided an EDTA blood sample, collected by research staff at the local sites and stored in -80 C freezers. DNA from cases was quantitated, normalized to a standard concentration, transferred to microplates in the NL and forwarded to University of Liverpool for processing. Participants from this study provided written consent for sharing of de-identified individual-level genotype and phenotype data; these data will be made available on dbGaP.

Statin-induced myopathy cases (n=4) evaluated at the Chicoutimi Hospital Lipid Clinic and ECOGENE-21 Clinical and Translational Research Center (Chicoutimi, Quebec, Canada) were also included in this study. The degree of myopathy was self-reported by subjects as part of a detailed questionnaire and from medical records. The clinical evaluation also included plasma CK and myoglobinuria assessment. Participants from this study did not provide consent for investigators to share their individual-level study data.

The Cardiovascular Health Study (CHS) is a prospective cohort study of risk factors for coronary heart disease and stroke in 5,888 adults aged 65 or older.^6, 7^ Subjects were followed with annual visits or telephone calls that assessed medical conditions, medication use, hospitalizations, and cardiovascular events. CHS participants with whole exome sequencing were selected as controls (n=584) if they used a statin during any study visit. Individual-level data on CHS participants are available on dbGaP (accession phs000667.v3.p1 ).

The Atherosclerosis Risk in Communities (ARIC) Study is an ongoing population-based cohort of 15,792 predominantly Caucasian and African-American males and females aged 45-64 years at baseline and selected using probability sampling from four United States communities (Forsyth County NC, Jackson MS, suburban Minneapolis MN, and Washington County MD).^8^ Participants were recruited in 1987-1989 to examine cardiovascular and pulmonary disease, patterns of medical care, and disease variation over time. Standardized physical examinations and interviewer-administered questionnaires were conducted at baseline (1987-1989), and at three triennial follow-up examinations (1990-1998). ARIC participants with whole exome sequencing were selected as controls (n=956) if they used a statin during any study visit. Participants from CHS sand ARIC comprised a pooled control group. Individual-level data on ARIC participants are available on dbGaP (accession phs000668.v3.p1).

**Recruitment and study populations: PREDICTION-ADR**

The Prediction-ADR cases (N=241) and controls (N=507) were recruited retrospectively and prospectively by the consortium‘s study centers in Uppsala (Sweden), Utrecht (the Netherlands), Dundee and Liverpool (UK) ^9^ based on previously published criteria (Supplementary Table 1).^9,10^ Statin myopathy cases were identified from population cohorts GoDARTS (N=36) and Genetics of Scottish Health Registry (GoSHARE) (N=53), from Clinical Practice Research Datalink (CPRD) (STAGE1 N=9, STAGE2 N=68) and Swedegene Biobank (N=27) that is linked to the adverse reaction registry at Medical Products Agency, Uppsala. In the UK, cases were also recruited by direct referrals from health professionals within NHS Trusts or primary care practice, by searching hospital database records or via the Yellow Card reporting system (MOLGEN study N=48). Cases were defined as patients on statin therapy with muscle symptoms and laboratory results spanning the myotoxicity phenotype categories SRM 3-5;^11^ all cases had a minimum measured creatine phosphokinase (CK) level greater than four times (>4x) the upper limit of normal (ULN). Controls were statin users for at least 90 days recruited from these same locations, who reported no symptoms of myopathy and had no associated CK level elevations. Clinical adjudication was undertaken by physicians and specialists. Multiple EU Research Ethics Committees (Tayside, Scotland; North West England, UK: Sefton, UK) approved the PREDICTION-ADR study and informed consent was obtained from all participants. Participants from this study did not provide consent for investigators to share their individual-level study data.

**Genotyping and variant calling: US-UK**

DNA samples were constructed into Illumina paired-end pre-capture libraries according to the manufacturer’s protocol. The complete protocol and oligonucleotide sequences are accessible from the Baylor College of Medicine Human Genome Sequencing Center (HGSC) website (<https://www.hgsc.bcm.edu/content/protocols-sequencing-library-construction>). Two, four or six pre-capture libraries were pooled together and then hybridized to the HGSC VCRome 2.1 design ^12^ (42Mb, NimbleGen) and sequenced in paired-end mode in a single lane on the Illumina HiSeq 2000 or the HiSeq 2500 platform. Illumina sequence analysis was performed using the HGSC Mercury analysis pipeline (<https://www.hgsc.bcm.edu/content/mercury>). Pooled samples were de-multiplexed using the Consensus assessment of sequence and variation (CASAVA) software. Reads were mapped to the Genome Reference Consortium Human Build 37(GRCh37) human reference sequence using BWA ^13^. Aligned reads were then recalibrated using GATK ^14^ along with BAM sorting, duplicate read marking, and realignment near insertions or deletions (indels). The Atlas2 suite was used to call single nucleotide variants (SNVs) and insertion-deletions (indels) ^15^.

Each SNV call was filtered based on the following criteria to produce a high-quality variant list: low SNV posterior probability (<0.95), low variant read count (<3), variant read ratio <0.25 or >0.75, strand-bias of more than 99% variant reads in a single strand direction, or total coverage less than 10-fold. All variant calls filtered by these criteria, and reference calls with less than 10-fold coverage, were set to missing. The variant call filters were the same for indels except a total coverage less than 30-fold was used for variant sites.

Variant-level quality control steps excluded variants outside the exon capture regions (VCRrome 2.1), monomorphic sites, missing rate >20%, mappability score <0.8, and mean depth of coverage >500-fold. Variants not meeting Hardy-Weinberg equilibrium expectations (*P*<5x10^-6^) in ancestry-specific groups were also excluded. Sample-level quality control metrics were calculated by cohort and ancestry group. A sample was excluded for missingness >20%, or if compared to the other samples it fell less than 6 standard deviations (SD) for mean depth, more than 6 SD for singleton count, or outside of 6 SD for heterozygote to homozygote ratio or Ti/Tv ratio. The mean depth of coverage was 78-fold.

**Genotyping and variant calling: PREDICTION-ADR**

All DNA samples underwent whole exome sequencing in 3 EU centres: cases were sequenced in the Centre for Genomic Research in Liverpool and at the University of Dundee, controls were sequenced in Liverpool, Dundee and Uppsala. Across all three genotyping centers for the PREDICTION-ADR study, the number of variants and depth of coverage were similar (**Supplemental Table 2**).

SureSelect QXT, XT and XT2 reagents (Agilent Technologies, Wokingham, UK) were used to perform fragmentation, end-repair, A-addition and adaptor ligation reactions to generate Illumina-compatible sequencing libraries in Dundee, Liverpool and Uppsala respectively. Hybridization capture enrichment of whole genome libraries was performed using the SureSelect v5 all-exon probe set, following manufacturer’s recommendations (<http://www.agilent.com/cs/library/usermanuals/Public/G9681-90000.pdf>). Pool of 10 to 12 libraries was sequenced on Nextseq500 or Hiseq2500 (Illumina Inc., San Diego, CA, USA) with version 2 TruSeq chemistry or Hiseq2500 and HiSeq 4000 (Illumina Inc., San Diego, CA, USA) or version 1 chemistry in Dundee and Liverpool respectively, generating 2 × 150bp paired end reads. The sequencing in Uppsala was made using HiSeq2500, paired-end 125bp read length and the v4 sequencing chemistry. Paired-end reads were aligned to the reference human genome (UCSC hg 19; <http://genome.ucsc.edu/>) using Burrows-Wheeler Alignment (BWA) mem algorithm (v0.7.12) ^13^. Duplicates were removed using Picard v1.85. Samtools 0.1.18-r580 was used to sort and index the aligned bam files. Base quality score recalibration as well as variant calling were performed using Genome Analysis ToolKit (GATK4 beta6) ^14^.

Quality control was performed using PLINK (v.1.90). Individuals with discordant sex information, missing rates >7%, outlying heterozygosity rate (3SD from the mean) and relatedness IBD > 0.185 were removed from the study. SNVs and indels were filtered for variant Hardy-Weinberg Equilibrium P-values < 1x10^-5^, genotyping rate < 5%, misingness between batches < 1x10^-5^ and association between controls from different centers 1x10^-4^. Ethnic outliers were identified and excluded using the MDS-plot feature in PLINK. All analyzed individuals were of Caucasian origin.
