# Supplementary material for: Pharmacogenomics of statin-related myopathy: Meta-analysis of rare variants from whole-exome sequencing
Source: PLoS One. 2019 Jun 26;14(6):e0218115. doi: 10.1371/journal.pone.0218115 (PMC6594672; doi:10.1371/journal.pone.0218115)
Supplement: S1 Table — (DOCX) [file pone.0218115.s002.docx]

**S1 Table.** Definition of statin-related myopathy.^11^

| Statin Related Myopathy (SRM) | Phenotype | Definition |
| --- | --- | --- |
| SRM 3 | Myopathy | CK elevation >4xULN <10xULN ± muscle symptoms, complete resolution on dechallenge |
| SRM 4 | Severe myopathy | CK elevation >10 x ULN<50x ULN with muscle symptoms, complete resolution on dechallenge |
| SRM 5 | Rhabdomyolysis | CK elevation >10xULN with evidence of renal impairment + muscle symptoms, or CK>50x ULN |
