# Supplementary material for: Pharmacogenomics of statin-related myopathy: Meta-analysis of rare variants from whole-exome sequencing
Source: PLoS One. 2019 Jun 26;14(6):e0218115. doi: 10.1371/journal.pone.0218115 (PMC6594672; doi:10.1371/journal.pone.0218115)
Supplement: S2 Table — (DOCX) [file pone.0218115.s003.docx]

S2 Table. Whole exome sequence metrics, PREDICTION-ADR

| Centre Average | Total number of reads | % mapped (with duplicates) | % at 20x coverage | % on target reads | % duplicates | No of variants |
| --- | --- | --- | --- | --- | --- | --- |
| Liverpool  (356 samples) | 75,289,026 | 98.36 | 89.85 | 56.59 | 23.98 | 44,372 |
| Uppsala  (349 samples) | 45,766,833 | 97.17 | 93.71 | 58.05 | 20.31 | 44,041 |
| Dundee  (336 samples) | 64,325,883 | 96.68 | 90.22 | 66.76 | 16.38 | 43,880 |
