# Supplementary figures and images for: Pharmacogenomics of statin-related myopathy: Meta-analysis of rare variants from whole-exome sequencing
Source: PLoS One. 2019 Jun 26;14(6):e0218115. doi: 10.1371/journal.pone.0218115 (PMC6594672; doi:10.1371/journal.pone.0218115)

**
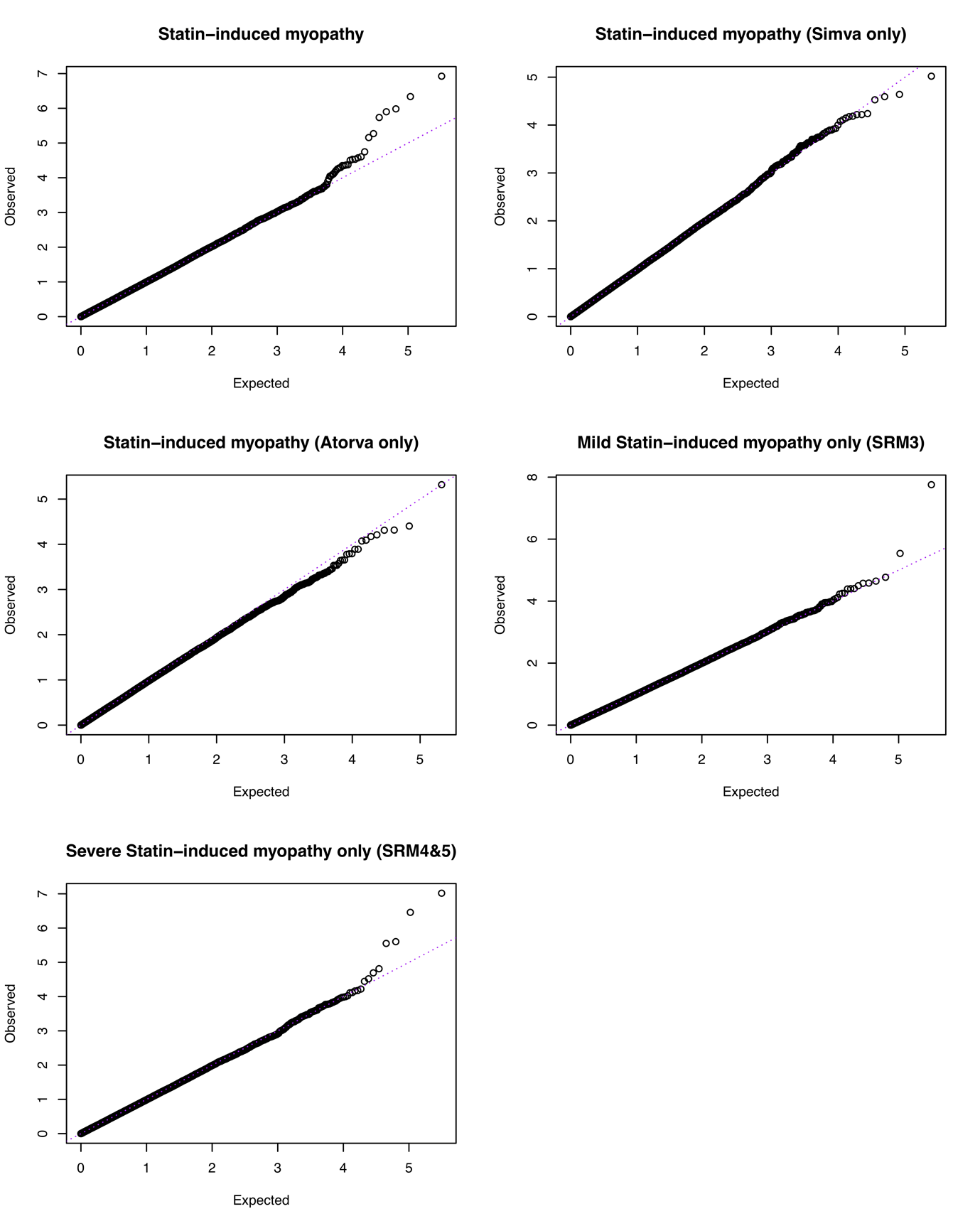
**

Supplement: S1 Fig — (DOCX) [file pone.0218115.s007.docx]

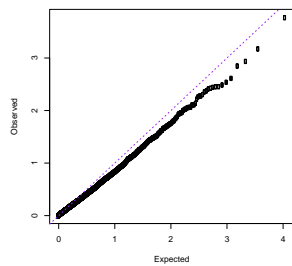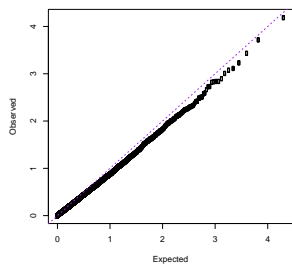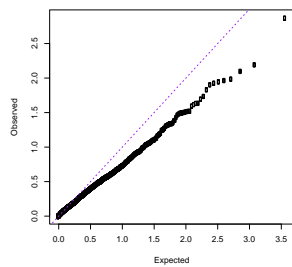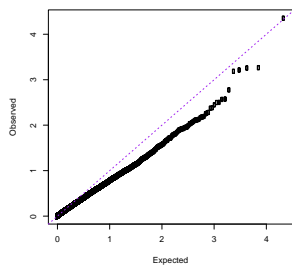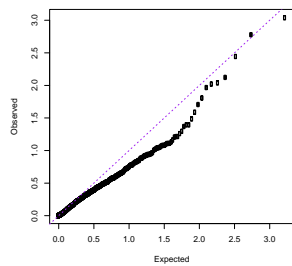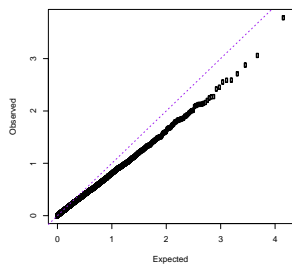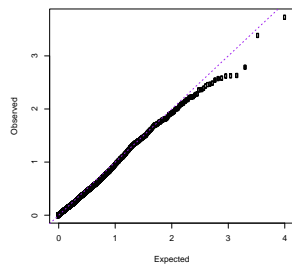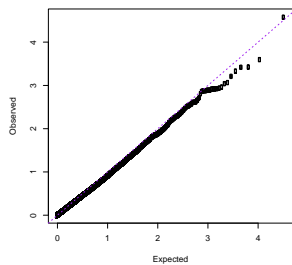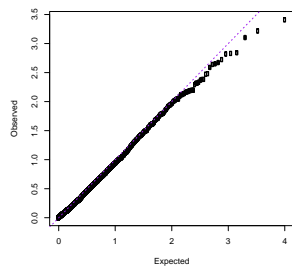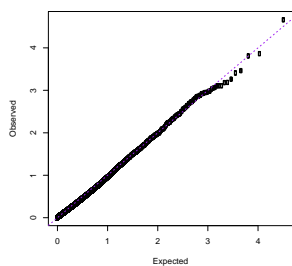

Supplement: S2 Fig — Columns: (1) predicted damaging variants, (2) all loss of function, nonsynonymous and missense. Rows: (1) all SRM, (2) simvastatin only, (3) atorvastatin only, (4) mild SRM, (5) severe SRM. (PDF) [file pone.0218115.s008.pdf]
